# Abacavir/Lamivudine plus Rilpivirine Is an Effective and Safe Strategy for HIV-1 Suppressed Patients: 48 Week Results of the SIMRIKI Retrospective Study

**DOI:** 10.1371/journal.pone.0164455

**Published:** 2016-10-11

**Authors:** Jesús Troya, Pablo Ryan, Esteban Ribera, Daniel Podzamczer, Victor Hontañón, Jose Alberto Terrón, Vicente Boix, Santiago Moreno, Pilar Barrufet, Manuel Castaño, Ana Carrero, María José Galindo, Ignacio Suárez-Lozano, Hernando Knobel, Miguel Raffo, Javier Solís, María Yllescas, Herminia Esteban, Juan González-García, Juan Berenguer, Arkaitz Imaz, GESIDA-8314 Study Group

**Affiliations:** 1 Hospital Universitario Infanta Leonor, Madrid, Spain; 2 Hospital Universitario Vall d’Hebrón, Barcelona, Spain; 3 Hospital Universitario de Bellvitge, Barcelona, Spain; 4 Hospital Universitario La Paz/IdiPAZ, Madrid, Spain; 5 Hospital Jerez de la Frontera, Jerez de la Frontera, Spain; 6 Hospital General Universitario de Alicante, Alicante, Spain; 7 Hospital Universitario Ramón y Cajal, Madrid, Spain; 8 Hospital de Mataró, Mataró, Spain; 9 Hospital Regional Universitario de Málaga, Málaga, Spain; 10 Hospital Universitario Gregorio Marañón, Madrid, Spain; 11 Hospital Clínico de Valencia, Valencia, Spain; 12 Hospital Universitario Infanta Elena, Huelva, Spain; 13 Hospital del Mar, Barcelona, Spain; 14 Fundación SEIMC-GESIDA, Madrid, Spain; Azienda Ospedaliera Universitaria di Perugia, ITALY

## Abstract

**Objectives:**

Based on data from clinical practice, we evaluated the effectiveness and safety of switching to abacavir/lamivudine plus rilpivirine (ABC/3TC+RPV) treatment in virologically suppressed HIV-1-infected patients.

**Methods:**

We performed a multicenter, non-controlled, retrospective study of HIV-1-infected patients who switched treatment to ABC/3TC+RPV. Patients had an HIV-RNA <50 copies/mL for at least 24 weeks prior to changing treatments. The primary objective was HIV-1 RNA <50 copies/mL at week 48. Effectiveness was analyzed by intention-to-treat (ITT), missing = failure and on-treatment (OT) analyses. The secondary objectives analyzed were adverse effects changes in renal, hepatic or lipid profiles, changes in CD4+ cell count and treatment discontinuations.

**Results:**

Of the 205 patients included, 75.6% were men and the median age was 49. At baseline, before switching to ABC/3TC+RPV, median time since HIV diagnosis was 13.1 years, median time with undetectable HIV-1 RNA was 6.2 years and median time of previous antiretroviral regimen was 3.1 years (48.3% patients were taking efavirenz and ABC/3TC was the most frequent backbone coformulation in 69.7% of patients). The main reasons for switching were drug toxicity/poor tolerability (60.5%) and simplification (20%). At week 48, the primary objective was achieved by 187 out of 205 (91.2%) patients by ITT analysis, and 187 out of 192 (97.4%) patients by OT analysis. The CD4+ lymphocyte count and CD4+ percentage increased significantly from baseline to week 48 by a median of 48 cells/μL (−50 to 189) and 1.2% (−1.3% to 4.1%), respectively, *P*<0.001. Thirty-eight adverse events (AE) were detected in 32 patients. Of these, 25 had no clear association with treatment. Three patients interrupted therapy due to AE. We observed a decrease in all lipid parameters, *P*<0.001, and a slight improvement in the glomerular filtration rate, *P*<0.01. Therapy was considered to have failed in 18 patients owing to virological failure (5 [2.4%]), toxicity/poor tolerability (4 [2%]), clinical decision (3 [1.5%]), loss to follow-up (3 [1.5%]), death (1 [0.5%]), and no clinical data (2 [1%]).

**Conclusions:**

The results of this study confirms that ABC/3TC+RPV is an effective, safe, and cost-effective option for the treatment of patients with virologically stable HIV-1 infection.

## Introduction

First-line combined antiretroviral therapies (cART) are currently highly effective [1–2]. However, toxicity in the short and long term still arises as a main concern for clinicians managing HIV infection.

Therapy regimens are sometimes switched to improve or maintain the patient’s quality of life and, in a few cases, to reduce treatment costs [3]. Strategies used when switching cART regimens are aimed at improving a patient’s quality of life without compromising treatment effectiveness. This is accomplished through the discontinuation of toxic drugs, the identification of poorly tolerated drugs and the management of drug interactions [4–6].

The combination of abacavir/lamivudine (ABC/3TC) is widely used in both clinical trials and real-world practice, especially when clinicians wish to avoid the toxicity of older nucleoside/nucleotide analogues, such as zidovudine, didanosine, stavudine and tenofovir (TDF) [7–9].

Based on the ECHO and THRIVE clinical trials [10–14] the second-generation non-nucleoside reverse-transcriptase inhibitor (NNRTI) rilpivirine (RPV) has been approved for treatment-naïve HIV-1-infected patients. Information about its combination with ABC/3TC in clinical trials is scarce, although favorable efficacy and safety results were observed in a small group of 35 treatment-naïve patients in the THRIVE clinical trial [12]. Clinical studies with RPV as a switching strategy have only been performed in combination with tenofovir/emtricitabine (TDF/FTC), demonstrating high efficacy, good tolerability profiles and high adherence [15–18].

The combination ABC/3TC+RPV might simultaneously address the issues of effectiveness, tolerability and ease of administration as an alternate therapy in routine clinical practice.

Given the lack of evidence on switching to ABC/3TC+RPV, it is important to test the feasibility of this strategy. We carried out a study to explore the effectiveness and safety of switching to a regimen comprised of a fixed-dose ABC/3TC and RPV in virologically suppressed HIV-1-infected patients.

## Methods

### Study design and patients

This was a multicenter, non-controlled and retrospective study of HIV-1-infected patients switching to ABC/3TC+RPV conducted in 13 hospitals in Spain. All included patients had to fulfill the following criteria: i) age ≥18 years, ii) documented HIV-1 infection, iii) switch from another therapy regimen to coformulated ABC/3TC (600/300 mg fixed-dose combination) and RPV (25 mg) once daily for any reason from March 2013 to March 2014 and iv) serum HIV RNA <50 copies/mL for at least 24 weeks before switching to ABC/3TC+RPV. Analysis was carried out in 2015, to ensure that the final patient included in 2014 had at least 48 weeks of follow up. Patients tested negative for HLA*B5701 before initiating ABC/3TC+RPV.

Eligible individuals were identified by a systematic search of the databases of each center. Data for the study was collected retrospectively from patient´s medical records, anonymized and entered into an electronic database. The information collected included: baseline demographic and HIV-related data, comorbidities, adverse events and laboratory results (blood count, biochemical parameters including lipid profile and liver and kidney function, CD4+ lymphocyte count, and HIV RNA), at baseline and thereafter according to the routine clinical protocols in each hospital *(every 12 to 24 weeks)*, reasons for treatment discontinuation in patients who stopped or changed therapy, and results of genotypic resistance testing after virological failure, when available.

The study protocol was approved by the Hospital Universitario Gregorio Marañón Ethics Committee with code SEI-RIL-2014-02, in accordance with the principles of the Declaration of Helsinki 2008. The study required local approval of several other Clinical Research Ethics Committees: Hospital Universitario Vall d´Hebrón Ethics Committee, Hospital de Mataró-Consorcio Sanitario del Maresme Ethics Committee, Hospital Universitario de Bellvitge Ethics Committee, Hospital Clínico Universitario de Valencia Ethics Committee and Ethics Committee of the province of Huelva.

Analysis with a view to scientific publication was based on anonymized routine clinical data, thus obviating the need for written informed consent.

### Outcomes

The primary objective was the percentage of patients who maintained virological suppression (HIV-1 RNA <50 copies/mL) after 48 weeks of treatment. This outcome was analyzed using intention to treat (ITT), missing = failure and on treatment (OT) analyses. Treatment was considered to have failed when any of the following events occurred: virological failure (VF), defined as two consecutive HIV RNA measurements >50 copies/mL or a single measurement of >50 copies/mL if treatment was changed afterwards; treatment interruption by patient; change of treatment regimen due to non-VF reasons and incomplete data. Viral load was evaluated 3 or 4 times during the 48-week period of the study to ascertain virologic control, according to the routine clinical protocols in each hospital. The secondary objectives were adverse events, changes in CD4 count, lipid, hepatic or renal profile changes, treatment discontinuations and reasons for discontinuations.

### Statistical analysis

All analyses were carried out using IBM SPSS Statistics for Windows®, Version 21.0 (SPSS, Chicago, IL, USA).

We analyzed the variables available at baseline and at 48 weeks. Variables that did not follow a normal distribution were described using the median and interquartile range (IQR). Nominal variables were described as numbers and percentages. Paired samples were compared using the *t* test (normally distributed variables) and the Wilcoxon test (non-normally distributed variables). Normality of the variables was tested with the Kolmogorov-Smirnov-Lilliefords test. The association between qualitative variables was assessed using the χ^2^ test when the sample was sufficiently large or Fisher's exact test when it was not. The 95% confidence intervals were calculated based on the Newcombe-Wilson hybrid score.

For the primary outcome (ITT analysis), we included all registered patients (incomplete or missing patients were defined as failures). We also performed an analysis excluding patients whose treatment was discontinued for non-virological reasons (OT analysis).

## Results

The study population comprised 205 patients. Baseline patient characteristics are summarized in Table 1. Men accounted for 76% of the sample, median age was 49 years, a quarter of patients had prior AIDS-defining conditions, and a fifth were co-infected with hepatitis C virus (HCV). At baseline, median CD4+ lymphocyte count was 667 cells/μL, and the median CD4+ lymphocyte nadir was 198 cells/μL.

**Table 1:** Baseline characteristics of the study patients.

	(n = 205)
**Age** (years); median (IQR)	49 (41–54)
**Gender**; n (%)	
Male	155 (75.6)
Female	50 (24.4)
**HIV risk factors**; n (%)	
Sexual relations between MSM	62 (30.2)
Heterosexual relations	65 (31.7)
IDU	61 (29.7)
Other/Unknown	17 (8.3)
**Baseline CD4**; median (IQR)	
CD4 count (cells/μ)	667 (471–870)
CD4%	31 (23–38)
**Nadir CD4** (cells/μ); median (IQR)	198 (87–288)
**AIDS diagnosis**; n (%)	54 (26.3)
**Time since HIV diagnosis** (years); median (IQR)	13.1 (6.1–18.7)
**Months since undetectable viral load** (<50 copies/mL); median (IQR)	74.8 (35.8–117.0)
**Comorbidities**; n (%)	
HBV (HbsAg +)	4 (2)
HCV (PCR +)	41 (20)
Hypertension	44 (22.5)
Diabetes mellitus	19 (9.3)
Dyslipidemia	59 (28.8)
Ischemic heart disease	12 (5.9)
Kidney disease	10 (4.9)
**Reasons for switching**; n (%)	
Drug toxicity/tolerability	124 (60.5%)
Regimen simplification	41 (20%)
Physician’s criteria*	23 (11.2%)
Cost savings	3 (1.5%)
Unknown reasons	14 (6.8%)
**Previous cART**; n (%)	
***Nucleos(t)ide***	
Abacavir	144 (70.2)
Tenofovir	53 (25.8)
Zidovudine	11 (5.4)
***Protease inhibitors***	
Atazanavir	24 (11.7)
Darunavir (monotherapy: 2)	22 (10.7)
Lopinavir (monotherapy: 2)	9 (4.4)
Other	8 (3.9)
***Non-nucleoside***	
Efavirenz	99 (48.3)
Etravirine	13 (6.3)
Nevirapine	6 (2.9)
Rilpivirine	4 (1.9)
***Integrase inhibitor***	
Raltegravir	3 (1.5)

MSM: men who have sex with men; IDU: injection drug user; AIDS: acquired immunodeficiency syndrome; HBV: hepatitis B virus; HCV: hepatitis C virus; cART: combined antiretroviral treatment.

* Potential drug interactions, better profile, a personal decision.

At baseline, before switching to ABC/3TC+RPV, median time from HIV diagnosis was 13.1 years, median time from first antiretroviral treatment was 9.6 years, median time of previous antiretroviral regimen was 3.1 years and median time with undetectable HIV-1 RNA was 6.2 years.

Prior to switching, 63 patients (30.7%) were taking a ritonavir-boosted protease inhibitor (bPI) and 122 patients (59.4%) a NNRTI (efavirenz, EFV, 48.3%). TDF was part of the previous backbone in 53 patients (25.8%) and ABC in 144 (70.2%). Of note, ABC/3TC was the most frequent nucleoside reverse transcriptase inhibitor (NRTI) backbone coformulation before switching (69.7%). The main reasons for switching were drug toxicity/tolerability in 124 cases (60.5%) and regimen simplification in 41 (20%). In the toxicity/tolerability group of patients, 82 (65.3%) were on EFV-based regimens and 25 (20.2%) on bPI-based regimens. Regimen simplification (doses per day or number of pills) resulted primarily from bPI and etravirine-based regimens (51.2% and 22%, respectively).

**Fig 1:**
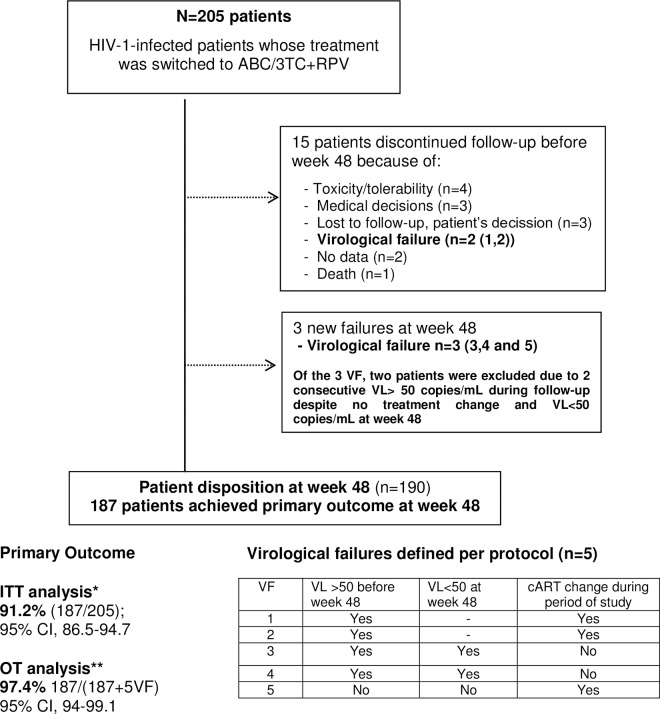
Study Flowchart. * ITT analysis: including all registered patients (incomplete or missing patients were included with patients whose treatment had failed). **OT analysis: excluding patients who discontinued treatment for non virological reasons. VF: virological failure, VL: viral load, cART: combined antiretroviral treatments.

The primary objective was observed in 187 out of 205 patients (91.2% [95% CI, 86.5–94.7]) by ITT analysis. When patients who had discontinued therapy for non-virological reasons were excluded, 187 out of 192 patients (97.4% [95% CI, 94–99.1]) reached the primary outcome per protocol (OT analysis) (Fig 1). Therapy failed in 18 patients (8.8%), including 15 who discontinued ABC/3TC+RPV. The reasons for discontinuation were toxicity/poor tolerability (4 [2%]), virological failure (2 [1%]), physician’s decision (3 [1.5%]), loss of clinical patient monitoring or patient’s decision (3 [1.5%]), death (1 [0.5%]), and no clinical data at the evaluation time point (2 [1%]). At week 48, five patients (2.4%) experienced protocol-defined virological failure. Of these, two continued with ABC/3TC+RPV after two consecutive low-level detectable HIV-1 RNA determinations (at weeks 6 and 24), and both had undetectable HIV-1 RNA at week 48. The other three patients had detectable HIV-1 RNA during the study period (two patients between weeks 4 and 8, and one patient at week 48). All three patients underwent resistance testing at the time of virological failure. Genotyping could not be performed in one patient due to an HIV-1 RNA level below 200 copies/mL, and in the other two patients, the resistance mutations found were as follows: NRTI mutations (M41L, M184V, T215F, K219Q) and NNRTI mutations (A98G, K103N, K238T) in one patient; and NRTI mutations (A62V, M184V), a NNRTI mutation (E138K), and a bPI mutation (L33V) in the other patient. Before switching to ABC/3TC+RPV, the available genotypic resistance tests did not show resistance mutations that would have compromised this treatment strategy. Therefore, no patients were excluded from the study for previous resistance mutations.

The median CD4+ lymphocyte count and median CD4+ percentage increased significantly from baseline to week 48 by a median of 48 cells/μL (−50 to 189) and 1.2% (-1.3% to 4.1%), respectively, *P*<0.001. In patients with a previous AIDS diagnosis (CDC stage C3), the CD4+ lymphocyte count and CD4+ percentage also increased significantly by a median of 50 cells/μL (−19 to 182.5) and 1.7% (−0.5% to 4.3%), respectively, *P*<0.001.

In this study cohort, 38 adverse events (AEs) were registered among 32 patients (15.6%) (Table 2). Clinical intervention was unnecessary for 33 of the events, and 25 resolved. Medication was discontinued in three (1.5%) of the five patients who required clinical intervention. The reasons for discontinuation were insomnia/depression, dizziness, and epigastralgia. Severe AEs were not related to medication, but moderate AEs could have been associated to the treatment in 3 patients. In 25 events, clinicians did not find a clear correlation between the event and antiretroviral therapy.

**Table 2:** Overview of adverse events (AEs).

**Summary of AE**	** **
Patients with ≥ 1 AE	32
Total number of AE	38
Patients with ≥1 Grade 3–4 AE	4
Total number of Grade 3 or 4 AE	5
Patients with serious AE	1
Total number of serious AE	1
Discontinuation due to AE	3*
Deaths	1
**Types of AE**	
Digestive	11
Neuropsychiatric	7
Infectious	7
Systemic	7
Dermatological	3
Cardiovascular	3
**Severity of adverse events**	
Severe (fatal or life-threatening)	1
Moderate (requires medical treatment or hospitalization)	5
Mild (symptoms do not require major medical intervention)	32
**Association with combination ABC/3TC+RPV**	
Related	2
Likely	5
Possible	3
Unlikely	3
Not related	25

* Insomnia/depression, dizziness, epigastralgia.

Median changes in lipid, hepatic, and renal profiles were analyzed at week 48 (Table 3). In this cohort of patients a small but statistically significant decrease in lipid levels were observed: total cholesterol (T-chol), with a percent change of −9.1% (median change, IQR, −18 [−47 to 3] mg/dL); low-density lipoprotein cholesterol (LDL-chol), −8.9% (−10.8 mg/dL [−43 to 9 mg/dL]); high-density lipoprotein cholesterol (HDL-chol), −7% (−4 mg/dL [−9 to 1 mg/dL]) and triglycerides (TG), −15% (−19 mg/dL [−51 to 12 mg/dL]); *P*<0.001 for all differences. There was no significant difference in lipid profiles in patients previously on a bPI-based regimen, but patients previously on an EFV-based regimen presented changes in T-chol, −15.4% (−29.6 mg/dL [−55.5 to −9 mg/dL], *P*<0.01), LDL-chol -14.6% (−20.5 mg/dL [−47 to 1.6 mg/dL], *P*<0.01), HDL-chol, −9.8% (−5 mg/dL [−9 to 0 mg/dL], *P*<0.01) and TG −20.3% (−28 mg/dL [−55 to 1.2 mg/dL], *P*<0.01).

**Table 3:** Laboratory values at baseline and at week 48.*

LIPID PROFILE		N	Median	P. 25	P. 75	p
Total cholesterol	Baseline (mg/dL)	192	202.9	178.0	233.1	
	At week 48 (mg/dL)	179	186.0	158.0	209.0	
	Change	167	−18.0	−47.0	3.0	**<0.001**^**$**^
	Percentage change	167	−9.1	−22.1	1.6	
High-density lipoprotein cholesterol	Baseline (mg/dL)	151	49.0	40.2	62.0	
	At week 48 (mg/dL)	152	46.0	39.0	58.0	
	Change	123	−4.0	−9.0	1.0	**<0.001**¥
	Percentage change	123	−7.0	−17.1	2.0	
Low-density lipoprotein cholesterol	Baseline (mg/dL)	144	123.7	93.5	153.5	
	At week 48 (mg/dL)	146	113.8	91.0	135.0	
	Change	118	−10.8	−43.0	9.0	**<0.001**^**$**^
	Percentage change	118	−8.9	−27.1	10.7	
Triglycerides	Baseline (mg/dL)	190	134.5	84	204	
	At week 48 (mg/dL)	180	108.0	75.5	163.5	
	Change	167	−19.0	−51.0	12.0	**<0.001**¥
	Percentage change	167	−15.0	−33.5	12.3	
**LIVER PROFILE**						
Aspartate aminotransferase	Baseline (IU/L)	164	25.0	20.0	34.0	
	At week 48 (IU/L)	155	24.0	20.0	31.0	
	Change	140	0.5	−4.0	4.0	0.953¥
Alanine aminotransferase	Baseline (IU/L)	189	25.8	17.0	40.0	
	At week 48 (IU/L)	181	25.0	18.0	40.0	
	Change	167	1.0	−9.0	8.0	0.931¥
Gamma-glutamyl transpeptidase	Baseline (IU/L)	167	39.0	23.0	71.4	
	At week 48 (IU/L)	165	29.0	21.0	53.0	
	Change	143	−7.0	−26.0	2.0	**<0.001**¥
Alkaline phosphatase	Baseline (IU/L)	177	87.0	68.0	108.0	
	At week 48 (IU/L)	151	75.0	62.0	90.0	
	Change	145	−12.0	−29.0	-1.0	**<0.001**¥
Bilirubin	Baseline (mg/dL)	154	0.5	0.4	0.70	
	At week 48 (mg/dL)	139	0.5	0.4	0.6	
	Change	122	0.0	−0.1	0.1	0.812¥
**RENAL PROFILE**						
Estimated glomerular filtration rate	Baseline	127	82.0	71.0	97.0	
	At week 48	147	89.4	74.6	99.6	
	Change	107	1.1	−4.8	10.0	**<0.01**¥
**CD4+ LYMPHOCYTE COUNT**						
	Baseline (cells/μL)	197	667	471	870	
Absolute CD4	At week 48 (cells/μL)	174	741	505	942	
	Change	167	48	−50	189	**<0.001**¥
	Baseline	199	31.0	23.7	38.0	
% CD4	At week 48	175	34.0	27.0	38.8	
	Change	170	1.2	−1.3	4.1	**<0.001**¥

^*^ Taking into account the retrospective nature of the study and according to the different routine clinical protocols in each hospital not all the patients had a baseline or 48-week lipid, hepatic or renal profiles.

^**$**^ p value Paired Samples T-test (parametric variables).

¥ p value Wilcoxon Signed-Rank test (nonparametric variables).

No changes were observed in liver profiles (aspartate aminotransferase, alanine aminotransferase, and bilirubin) at week 48, except for a slight decrease in gamma-glutamyl transpeptidase and alkaline phosphatase levels. This decrease was especially significant in the group of patients previously on an EFV-based regimen, median gamma-glutamyl transpeptidase of −18.5 IU/L (−41.7 to −7 IU/L) and a median alkaline phosphatase of −16.4 IU/L (−35 to −5 IU/L), *P*<0.01.

The estimated glomerular filtration rate (eGFR; Modification of Diet in Renal Disease equation) increased by a median (IQR) of 1.1 mL/min/1.73 m^2^ (−4.8 to 10) by week 48, *P*<0.01. Most of the patients who attained an increase in eGFR were taking TDF before switching (n = 55) and these patients had a median increase in eGFR of 7 mL/min/1.73 m^2^ (−5.8 to 13.1) at the end of follow-up, *P*<0.04. There were no significant changes in eGFR in patients previously on EFV or bIP-based regimens.

## Discussion

To our knowledge, there is little data showing that switching to ABC/3TC+RPV is effective, safe, and well tolerated in virologically suppressed HIV-1-infected patients. Previous studies have demonstrated that when switched from EFV+FTC/TDF, RPV+FTC/TDF is not inferior in terms of efficacy, has a better tolerability profile (resolving neuropsychiatric toxicity), and patients have a high adherence to this regimen [17, 18]. In terms of effectiveness, our findings also revealed a high virological suppression rate (above 90% at 48 weeks of follow-up), which in fact is similar to other reported studies on treatment switching [15–20].

Of the 205 patients included, 18 experienced treatment failure, although only five experienced protocol-defined virological failure during the 48 weeks of the study period. Interestingly, two of those patients presenting as virological failures had two consecutive HIV-1 RNA values between 50 and 200 copies/mL and later achieved and maintained HIV-1 RNA <50 copies/mL without having to modify their treatment. Resistance testing performed on samples from the other three patients who experienced virological failure revealed mutations that may have compromised the efficacy of the regimen in two cases. A high proportion of patients maintained an undetectable viral load at week 48 after the switch, probably because of the excellent tolerability profile of ABC/3TC+RPV and the good adherence motivated by the low pill burden and once-daily regimen.

We documented a median increase of 48 cells/μL (7.2%) in the CD4+ lymphocyte count but only a 1.2% increase in the percentage of CD4+ cells. Immune function was largely unchanged given the small and clinically insignificant change in CD4%. The absolute CD4 change may have been driven by increases in white blood cells or total lymphocyte count. Further follow-up would be needed in order to observe potential clinically significant changes.

The frequency of adverse events was low (15.6%). The attending physicians considered most events to be unrelated to the study medication, and most AES resolved. Only three patients discontinued the regimen owing to adverse events. The low number of discontinuations due to poor tolerability indicates that the regimen was well tolerated.

A small irrelevant decrease was observed in the serum concentrations of all fasting lipids. This observation was more evident in the group of patients with a previous EFV-based regimen, comprising nearly half of the patients who switched to RPV+ABC/3TC. This finding is similar to findings described in other studies with EFV, in naïve or treated patients [21, 22]. No significant changes were observed in the subgroup of previous bPI-based regimens despite findings in other studies [23].

Additionally, serum biochemistry analysis revealed a small but statistically significant decrease in alkaline phosphatase and gamma-glutamyl transpeptidase levels, especially among the group of patients with a previous EFV-based regimen. EFV is known to produce enzyme induction in the liver through CYP3A4 and has been described in the EFV data sheet in 7% of the patients who receive 600 mg of this drug [24].

Despite a significant median increase in eGFR of 1.1 mL/min/1.73 m^2^, no clinical relevance could be established. This increase is much more striking in the subgroup of patients who switched to ABC/3TC+RPV from a regimen containing TDF with a median increase in the eGFR of 7 mL/min/1.73 m^2^. This finding may reflect either a decrease in the inhibition of the tubular secretion of creatinine or an improvement in renal function after removing TDF from the regimen [25, 26]. Future availability of tenofovir-alafenamide (TAF) resulting in a better renal profile could avoid the potential renal damage observed in some patients using TDF [27].

Our study has several limitations. First, it was a retrospective study. Second, there were differences in clinical protocols and visit timetables between the study centers. It also lacked a comparative arm.

Nevertheless, our findings are relevant, because ABC/3TC+RPV proved to be highly effective in virologically suppressed HIV-1-infected patients. Therefore, this combination could potentially form the basis for a switching strategy, especially when TDF cannot be taken because of toxicity issues, resistance, or clinical reasons. Safety is yet another key advantage of ABC/3TC+RPV. For example, adverse events are infrequent and a regimen free of TDF has potential long-term benefits for the kidneys and bones.

In addition, this switching strategy could be cheaper than the most common antiretroviral regimens that include protease and integrase inhibitors. These savings can have a significant economic impact on the hospital drug expenses.

In summary, the results of the SIMRIKI study support the use of ABC/3TC+RPV as an effective, safe, well-tolerated and cost-effective switching strategy in patients with virologically stable HIV-1 infection.
