# Correction: Abacavir/Lamivudine plus Rilpivirine Is an Effective and Safe Strategy for HIV-1 Suppressed Patients: 48 Week Results of the SIMRIKI Retrospective Study

**DOI:** 10.1371/journal.pone.0172184

**Published:** 2017-02-09

**Authors:** Jesús Troya, Pablo Ryan, Esteban Ribera, Daniel Podzamczer, Victor Hontañón, Jose Alberto Terrón, Vicente Boix, Santiago Moreno, Pilar Barrufet, Manuel Castaño, Ana Carrero, María José Galindo, Ignacio Suárez-Lozano, Hernando Knobel, Miguel Raffo, Javier Solís, María Yllescas, Herminia Esteban, Juan González-García, Juan Berenguer, Arkaitz Imaz, GESIDA-8314 Study Group

The affiliation of the author Juan Berenguer is incorrect. The correct affiliation is: Hospital Universitario Gregorio Marañón, Madrid, Spain.
